# The Cyclophilin *ROC3* Regulates ABA-Induced Stomatal Closure and the Drought Stress Response of *Arabidopsis thaliana*

**DOI:** 10.3389/fpls.2021.668792

**Published:** 2021-05-25

**Authors:** Huiping Liu, Jianlin Shen, Chao Yuan, Dongxue Lu, Biswa R. Acharya, Mei Wang, Donghua Chen, Wei Zhang

**Affiliations:** ^1^Key Laboratory of Plant Development and Environmental Adaption Biology, Ministry of Education, School of Life Science, Shandong University, Qingdao, China; ^2^College of Natural and Agricultural Sciences, University of California, Riverside, Riverside, CA, United States

**Keywords:** *ROC3*, abscisic acid, stomatal closure, drought stress, anion channel, reactive oxygen species, catalase

## Abstract

Drought causes a major constraint on plant growth, development, and crop productivity. Drought stress enhances the synthesis and mobilization of the phytohormone abscisic acid (ABA). Enhanced cellular levels of ABA promote the production of reactive oxygen species (ROS), which in turn induce anion channel activity in guard cells that consequently leads to stomatal closure. Although Cyclophilins (CYPs) are known to participate in the biotic stress response, their involvement in guard cell ABA signaling and the drought response remains to be established. The *Arabidopsis thaliana* gene *ROC3* encodes a CYP. Arabidopsis *roc3* T-DNA mutants showed a reduced level of ABA-activated S-type anion currents, and stomatal closure than wild type (WT). Also, *roc3* mutants exhibited rapid loss of water in leaf than wild type. Two complementation lines of *roc3* mutants showed similar stomatal response to ABA as observed for WT. Both complementation lines also showed similar water loss as WT by leaf detached assay. Biochemical assay suggested that *ROC3* positively regulates ROS accumulation by inhibiting catalase activity. In response to ABA treatment or drought stress, *roc3* mutant show down regulation of a number of stress responsive genes. All findings indicate that *ROC3* positively regulates ABA-induced stomatal closure and the drought response by regulating ROS homeostasis and the expression of various stress-activated genes.

## Introduction

Drought stress causes a major constraint on plant growth, development, and productivity (Langridge and Reynolds, 2015). Stomata are surrounded by pairs of specialized epidermal cells termed guard cells, and which are essential for controlling gas exchange (carbon dioxide and oxygen) and water loss. Plants have the ability to adapt to drought stress by regulating stomatal closure (Agurla et al., 2018). The turgor and volume of the pair of guard cells determine the extent of the stomata's aperture (Schroeder et al., 2001). Various environmental cues regulate stomatal movement, but at the cellular level the most critical factor is the phytohormone abscisic acid (ABA) (Raghavendra et al., 2010; Kollist et al., 2014; Osakabe et al., 2014; Murata et al., 2015). During moisture deficiency, ABA synthesis in the leaf vasculature is enhanced and the hormone is transported to guard cells (Seo and Koshiba, 2011; Munemasa et al., 2015). The accumulation of ABA activates S-type anion channels in guard cells, resulting in an efflux of anions and a consequent decreases in guard cells' turgor, which eventually induces stomatal closure (Li et al., 2000; Wang et al., 2001; Vahisalu et al., 2008).

The simultaneous functions of many signaling molecules in guard cells provide stomatal defense response against drought stress. Reactive oxygen species (ROS) are major secondary messengers which play a crucial role in ABA-triggered stomatal closure (Pei et al., 2000; Zhang et al., 2001; Kwak et al., 2003; Bright et al., 2006; Miao et al., 2006; Murata et al., 2015). Drought stress induces the accumulation of ABA in guard cells, subsequently that leads to the activation of NADPH oxidase and the promotion of ROS production. The elevated level of ROS can also enhance the level of NO and cytosolic calcium. As a result, plasma membrane localized anion channels in guard cells are activated, consequently that leads to the efflux of anions and stomatal closure (Pei et al., 2000; Schroeder et al., 2001; Kwak et al., 2003; Mori et al., 2006; Munemasa et al., 2015). However, oxidative stress is induced in response to high concentration of ROS that causes injuries in plant cells. Hence plant cells are equipped with the antioxidant systems composed of superoxide dismutase (SOD), glutathione peroxidase (GPX), ascorbate peroxidase (APX), catalase (CAT), and non-enzymatic antioxidants to control the homeostasis of ROS (Willekens et al., 1997; Corpas et al., 2001; Mittler, 2002; Apel and Hirt, 2004; Nyathi and Baker, 2006; Palma et al., 2009; Jannat et al., 2011). However, uncontrolled action of antioxidant systems could reduce appropriate levels of ROS that function as secondary messengers, which would negatively impact the ABA and/or drought signal transduction systems. It is unknown how antioxidant systems are regulated during ABA and drought signaling to maintain the suitable levels of ROS in plants.

The Cyclophilins (CYPs) belong to a large class of proteins, referred to as the immunophilins. This class of protein is widely distributed across both prokaryotes and eukaryotes (Handschumacher et al., 1984; Wang et al., 2005; Kim et al., 2012). Plant CYPs have been shown to participate in a diversity of physiological processes, including protein folding, transcriptional regulation, and stress response (Santos and Park, 2019). For example, the rice protein CYP18-2, regulates the transcription and post-transcriptional modification of a number of stress-related genes (Lee et al., 2015), and over-expression of rice *CYP19-4* could enhance the plant cold tolerance as well (Yoon et al., 2016). There are 29 genes predicted to encode CYP or CYP-like proteins in the *Arabidopsis thaliana* genome (He et al., 2004). The ROC3 (also known as AtCYP19-1) has been shown to boost the plant's ability to withstand infection by the pathogen *Pseudomonas syringae* (Pogorelko et al., 2014). The present experiments were designed to explore whether ROC3 also plays any roles in drought stress tolerance and ABA signaling in *A. thaliana*.

## Materials and Methods

### Plant Materials and Growing Conditions

The *A. thaliana*, Columbia-0 (Col-0) was used as the wild-type (WT) for all experiments in this study. Seeds of the two T-DNA insertion mutants, *roc3-1* (SALK_063724c) and *-2* (SALK_095698c), in Columbia-0 background, were obtained from Arabidopsis Biological Resource Center (http://abrc.osu.edu/). The homozygous status of both T-DNA mutants was verified using a PCR assay (primer sequences are given in Supplementary Table 1). Seeds were surface sterilized in 75% v/v ethanol for 3 min, followed by 1 min in 95% v/v ethanol and then dried in air. The sterilized seeds were plated on half strength Murashige and Skoog (1962) medium (1/2 MS) containing 0.7% w/v agar. The plates were kept in the dark for 3 days at 4°C for vernalization, then transferred to a controlled growth chamber (~70% relative humidity) for 7–10 days, with 16 h photoperiod (100 μmol m^−2^s^−1^ fluorescent lamp light) and day/night temperature regime of 22 ± 1°C/16 ± 4°C. Thereafter, the seedlings were transplanted to pots containing mixture of soil and vermiculite (2:1 (v/v), and transferred back to the growth chamber.

### Generation of Transgenic Plants Harboring p*ROC3::GUS* and GUS Assay

The *ROC3* native promoter, 1854 bp fragment upstream of the initiation codon (from CTTCTCACAT to AAAAAAAGAA of WT genomic DNA), was PCR-amplified from WT genomic DNA using the primer pair *ROC3-GUS-F/R* (sequences are given in Supplementary Table 1) and the amplicon was inserted into the HindIII and SmaI cloning sites of the p*Cambia-ubiGUS* vector to generate the construct p*ROC3::GUS*. The recombinant plasmid was introduced into *Agrobacterium tumefaciens* strain GV3101, then transformed into *Arabidopsis* via *Agrobacterium*-mediated transformation using the floral dip technique (Clough and Bent, 1998). p*ROC3::GUS* activity was detected in seedling (2-week-old), root (2-week-old), leaf (2-week-old), flower (6-week-old), silique (8-week-old), and guard cells (4-week-old) of transgenic plants. The described plant tissues were immersed for ~3 h at 37°C in 50 mM sodium phosphate buffer (pH 7.2) containing 2 mM X-Gluc, 2 mM K_3_Fe(CN)_6_, 2 mM K_4_Fe(CN)_6_, 0.1% v/v Triton X-100, and 10 mM EDTA. Subsequently, the samples were incubated in absolute ethanol to remove chlorophyll and then inspected under a stereomicroscope (SZX2-ILLT; OLYMPUS, Tokyo, Japan). For ABA treatment tests, the samples were treated with 50 μM ABA or absolute ethanol (solvent control) respectively for 2.5h before staining. Relative GUS activity was quantified from three independent biological replicates using ImageJ open source software (v. 1.37, https://imagej.nih.gov/ij/).

### Subcellular Localization Assay of *ROC3*

To generate the construct p*35S::ROC3-GFP*, the full length cDNA of *ROC3* was PCR-amplified from WT cDNA using the primer pair *ROC3-GFP-F/R* (Supplementary Table 1) and the amplicon inserted into the BamHI and SalI sites of the *pBI221-GFP* vector, with the GFP in the carboxyl terminus (Lin et al., 2009). The plasmids p*35S::GFP*, p*35S::ROC3-GFP* and p*35S::AHL22-RFP* (used as a nuclear localization marker) (Xiao et al., 2009) were isolated with NucleoBond® Xtra Midi Kit (Macherey-Nagel, Germany) and transfected into Arabidopsis mesophyll protoplasts (Sheen, 2001). The protoplast cultures were incubated in the dark for 16 h at 23°C and then fluorescence was assessed using laser scanning confocal microscopy (LSM880; Carl Zeiss, Oberkochen, Germany). The GFP signal was detected at excitation wavelengths of 488 and emission between 495 and 540 nm. The excitation of RFP was conducted at 543 nm, with emission being captured between 580 and 620 nm.

### Stomatal Aperture Assay

The bioassay for stomatal aperture was performed as reported by Li et al. (2016) with a slight modification. In short, the leaves of 4-week-old plants were excised and incubated in closure buffer (20 mM KCl, 1 mM CaCl_2_, 5 mM MES-KOH, pH 6.15) at 23°C for 2.5 h in light (100 μmol m^−2^s^−1^ fluorescent lamp light), then added ABA (1, 10, 50 μM), 20 mM 3-amino-1,2,4-triazole (AT, an inhibitor of catalase) (Jannat et al., 2011), 100 μM H_2_O_2_ or absolute ethanol (solvent control) respectively for an additional 2.5 h in the same incubation place and condition. Subsequently, abaxial epidermal strips were peeled off and immediately photographed by a light inverted microscope. Stomatal aperture width and length were measured by the open access software ImageJ (v1.37, https://imagej.nih.gov/ij/). Each experiment included at least three biological replicates, with no fewer than 60 guard cells that were measured per each sample. The Student′s *t*-test was used to determine whether differences between mean values were statistically significant.

### Drought Stress and Water Loss Experiments

Seedlings were potted into the soil/vermiculite mixture and grown for about 4 weeks in well-watered condition, then water was withheld for 3 weeks. At the end of the treatment, the plants were re-watered over a 3 d period and photographed. Water loss assay from detached rosette leaves was sampled from 4-week-old well-watered plants. Detached rosette leaves were placed on filter paper in the light at room temperature and measured by weighing the leaves every 30 min over a 3 h period to measure the rate of water loss. The entire experiment was performed at least three biological replicates in the controlled growth chamber under the same light condition.

### qRT-PCR Assay

Two-week-old seedlings grown on a half-strength Murashige-Skoog (1/2 MS) medium (0.7% w/v agar) were incubated in liquid 1/2 MS for 24 h in a growth chamber (~70% relative humidity, 16 h photoperiod, 100 μmol m^−2^s^−1^ fluorescent lamp light, 22 ± 1°C/16 ± 4°C). Then the first set of seedlings was transferred to 1/2 MS liquid medium containing ABA (final concentration 50 μM); and a second set of seedlings was exposed on filter paper for drought treatment. Both ABA and drought treated seedlings, together with their control samples, were placed in the controlled growth chamber. Treated samples and their controls were collected at the same time after treatments, and snap-frozen in liquid nitrogen. RNA extracted from the frozen seedlings using the TRIzol reagent (Sigma–Aldrich, St. Louis, United States), was used to synthesize cDNA using a 5X All-In-One MasterMIX (with an AccuRT Genomic DNA Removal Kit (ABM, Canada). A quantitative real time (qRT)-PCR assay was performed using the FastStart Universal SYBR Green master mix (Roche, Basel, Switzerland) and the *ROC3-qRT-F/R* and *ACTIN2-qRT-F/R* primer pairs (Supplementary Table 1). qRT-PCR was also performed to quantify the transcript abundances of various genes encoding ROS signaling enzymes and stress-responsive proteins using specific primer pairs (Supplementary Table 1). All the quantitative analyses included three independent biological replicates, and each replicate contained three technical duplicates. *ACTIN2* used as the internal reference. The reaction steps were firstly pre-incubated at 95°C for 300 s, then ran 40 cycles with 95°C for 15 s, 58°C for 15 s, and 72°C for 20 s. CFX Connect^TM^ Real-Time PCR Detection System (Bio-Rad, California, U.S.A.) was used and the data was quantified by the ΔΔCt method.

### Complementation of the *roc3* Loss-of-Function Mutants

The *ROC3* open reading frame was PCR-amplified from Col-0 cDNA using the primer pair *ROC3-C-F/R* (Supplementary Table 1). The amplicon was inserted into the SmaI and SacI cloning sites of p*ROC3::GUS* vector to generate the transgene construct p*ROC3::ROC3. Agrobacterium tumefaciens* (strain GV3101) was transformed using this construct. The *roc3-1* and *roc3-2* mutants were transformed via *Agrobacterium* mediated transformation using the floral dip method (Clough and Bent, 1998). Transformants were selected on 1/2 MS Agar medium containing 30 mg/L hygromycin. *ROC3* complementation lines generated from *roc3-1* and *roc3-2* mutants were named as C-1 and C-2, respectively.

### Guard Cell Isolation and Electrophysiology

*A. thaliana* guard cell protoplasts were isolated as described previously (Zhang et al., 2008) with slight modifications. In brief, 10–12 rosette leaves from 4-week-old plants were cut off, and epidermal strips were peeled off. Then, the peeled epidermal strips were blended in a blender filled with 750 mL cold distilled water for 30 s, and filtered through a 100-μm nylon mesh and placed in a 10 mL beaker filled with 2 mL enzyme solution I [0.7% Cellulysin cellulase, 0.1% PVP-40, 0.25% BSA in 55% basic solution (5 mM MES, 0.5 mM CaCl_2_, 0.5 mM MgCl_2_, 0.5 mM ascorbic acid, 10 μM KH_2_PO_4_, 0.55 M sorbitol, pH 5.5)]. The beaker was placed in a water bath shaker and shook at 80 rpm for 30 min at 28°C, then 2 mL basic solution was added to enzyme solution I and shook for another 10 min. After that, the strips were filtered through a 100-μm nylon mesh and placed in beaker containing 2 mL enzyme solution II (1.5% Onuzuka cellulase RS, 0.01% cellulase Y-23, 0.25% BSA in 100% basic solution), and shaking was continued at 60 rpm for at least 15 min. Subsequently, the materials were mixed by pipetting up and down with a 1-mL pipette and filtered through a 30-μm nylon mesh. The protoplasts were centrifuged at 800 rpm for 5 min and washed twice with basic solution.

The whole-cell mode patch-clamp electrophysiology tests were carried out as described in the previous articles (Schroeder and Hagiwara, 1989; Pei et al., 1997; Vahisalu et al., 2008; Acharya et al., 2013). For anion current recordings, the bath solution contained 2 mM MgCl_2_, 30 mM CsCl, 1 mM CaCl_2_, 10 mM MES-Tris (pH 5.6) and the osmolarity of this solution was adjusted to 480 mOsm with sorbitol. The pipette solution contained 150 mM CsCl, 2 mM MgCl_2_, 6.7 mM EDTA, 3.35 mM CaCl_2_, and 10 mM HEPES (pH 7.5), the osmolarity of this solution was adjusted to 500 mOsm with sorbitol. ATP (10 mM Mg-ATP) and GTP (10 mM) were added to it before experiments. The whole-cell currents were recorded using the Axopath-200B amplifier (Molecular Devices, Downingtown, PA, USA) after the whole-cell configuration was achieved. The holding potential was +30 mV and voltage steps were applied from −145 to +35 mV with +30 mV increments, and each test voltage lasted 60 s. For ABA treatment tests, guard cell protoplasts were treated with 50 μM ABA for at least 1h before measurement. In order to obtain the currents and draw the current density voltage plots, pCLAMP software (version10.2; Axon Instruments, Sunnyvale, CA, USA) and SigmaPlot 12.0 (Systat Software, Richmond, CA, USA) were used respectively.

### Quantification of Guard Cell ROS and Leaf H_2_O_2_ and Catalase Levels

Quantification ROS content of both WT and *roc3* guard cells was performed using the fluorescent dye CM-H_2_DCFDA (Thermo Fisher, Waltham, MA, USA) as described previously with slight modifications (Miao et al., 2006; Zhang et al., 2011). In order to open the stomata, abaxial epidermis strips were prepared from the leaves of 4-week-old plants and immersed in 1 mM CaCl_2_, 20 mM KCl, 5 mM MES-KOH (pH 6.15) for 2.5 h in the light; then added 50 μM ABA or absolute ethanol as solvent control and the incubation continued for an additional 2.5 h. At the end of this period, the epidermal peels were transferred to aqueous 50 μM CM-H_2_DCFDA for 10 min in the dark, then rinsed at least three times in distilled water to remove excess dye. The H_2_DCFDA fluorescence was captured using laser scanning confocal microscope and inverted fluorescence microscope (TI-1; NIKON, Tokyo, Japan), and the fluorescence intensity was quantified by using ImageJ software.

To measure the leaf H_2_O_2_ content, the leave samples (4-week-old) were first weighed and immersed in liquid buffer (20 mM KCl, 1 mM CaCl_2_, 5 mM MES-KOH, pH 6.15) for 2.5 h, then added 50 μM ABA or absolute ethanol (solvent control), and the incubation continued for a additional 2.5 h. Snap-frozen leaf material was ground to a powder and processed using a commercial H_2_O_2_ content determination kit (Comin, Suzhou, China) according to the supplier's protocol. Finally, 200 μL aliquots of these reaction mix were pipetted into plates, and their absorbance at 415 nm was measured; H_2_O_2_ content was calculated according to the formula: H_2_O_2_ (μmol/g) = 2.67 × (ΔA − 0.0006) ÷ W (ΔA = A_sample_ − −*A*_blank_, W:sample weight).

Catalase assay were performed using a CAT detection kit (Comin, Suzhou, China). The samples were treated as described above in the method of H_2_O_2_ content assay_._ Then catalase extraction was performed according to the manufacturers' instruction. To quantify catalase, 10 μL aliquot of the extract added to 190 μL of Comin catalase reaction solution in a 96-well UV plate. The reactions' absorbance at 240 nm was recorded immediately (A1) and subsequently after 1 min (A2). Catalase activity was calculated on the basis of the formula: CAT (nmol/min/g) = 918 × ΔA ÷ W (ΔA = A1 − −A2, W:sample weight).

## Results

### The Response of *ROC3* to ABA Treatment and Drought Stress

**Figure 1:**
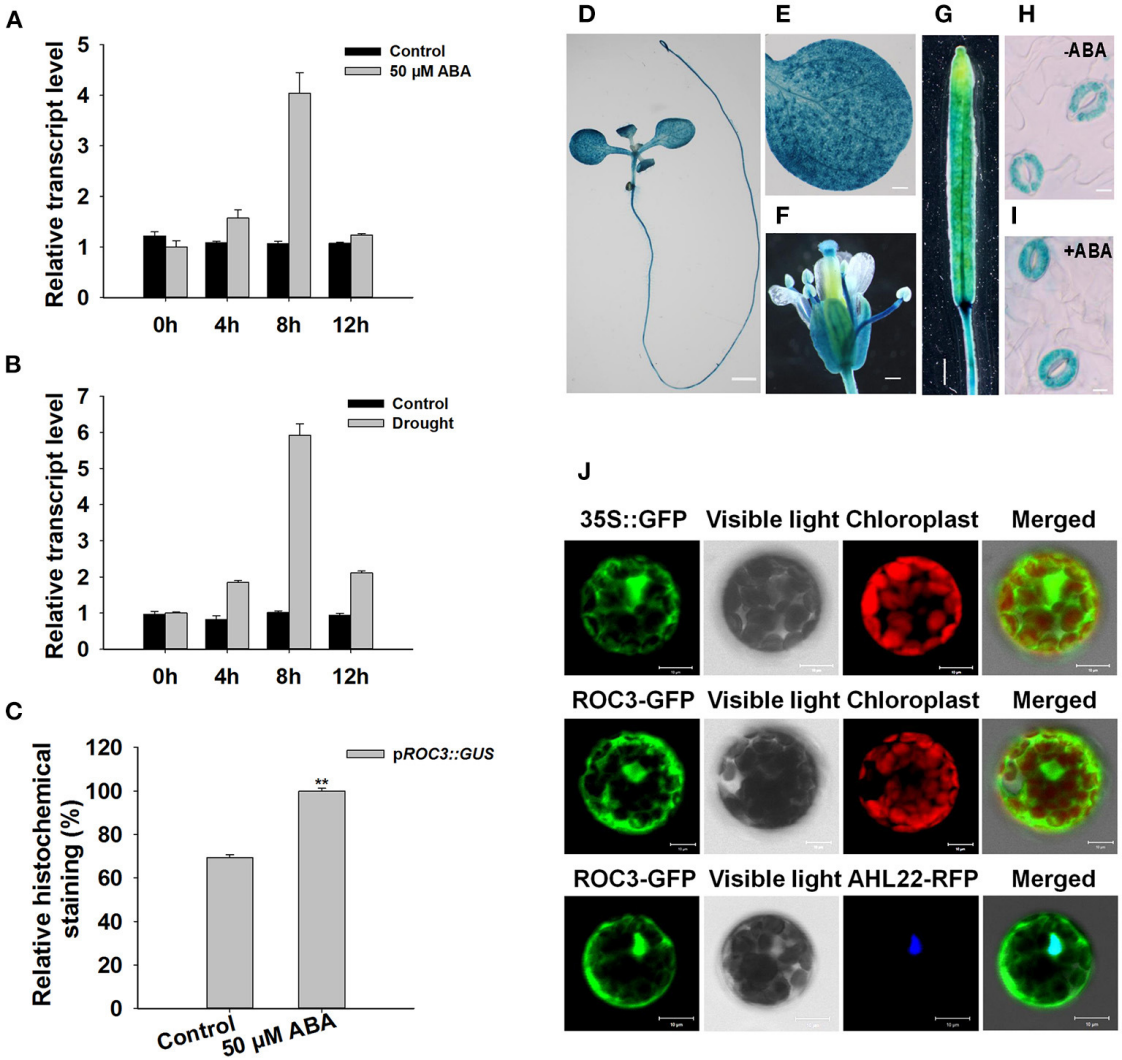
Transcriptional and expression profiling of *ROC3*. **(A,B)** Relative transcript abundances assessed using qRT-PCR in seedlings subjected to **(A)** ABA treatment, **(B)** drought stress. **(C)** Relative GUS activity of p*ROC3::GUS* in guard cells. Error bars represent the SE (*n* = 30), **: means differed significantly from control (*P* < 0.01). The experiments were repeated three times with similar results. **(D–I)** GUS staining reveals the tissue expression of p*ROC3::GUS* transgene in **(D)** the whole seedling (bar: 1.5 mm), **(E)** the leaf (bar: 0.3 mm), **(F)** the flower (bar: 0.3 mm), **(G)** the silique (bar: 1 mm) and **(H,I)** guard cells in plants **(H)** not exposed to ABA (bar: 10 μm), **(I)** exposed to 50 μM ABA (bar: 10 μm). **(J)** The topological expression of p*35S::GFP*, p*35S::ROC3-GFP*, and p*35S::AHL22-RFP* in protoplasts (bar: 10 μm).

Gene expression profiles of *ROC3* showed that the gene was induced by both ABA treatment and drought stress (Figures 1A,B). To test spatial expression of *ROC3* promoter, histochemical analysis was performed in transgenic plants expressing p*ROC3::GUS* construct. GUS expression was observed in seedling, leaf, flower, and silique (Figures 1D–G). Besides, *ROC3* promoter was mildly induced in the guard cells of plants treated with ABA (Figures 1C,H,I). Transient expression of GFP-tagged ROC3 (p*35S::ROC3-GFP*) in Arabidopsis mesophyll protoplasts showed that ROC3 located in both the cytoplasm and the nucleus (Figure 1J). To further confirm the nucleus localization of ROC3, the p*35S::ROC3-GFP* was co-transformed into protoplasts with the p*35S::AHL22-RFP* (AHL22, a known nucleus protein), and the GFP and RFP signals substantially overlapped in the nucleus (Figure 1J).

### The Stomatal Closure in *roc3* Is Hyposensitive to ABA and *roc3* Mutants Show a Lower Tolerance to Drought Stress

**Figure 2:**
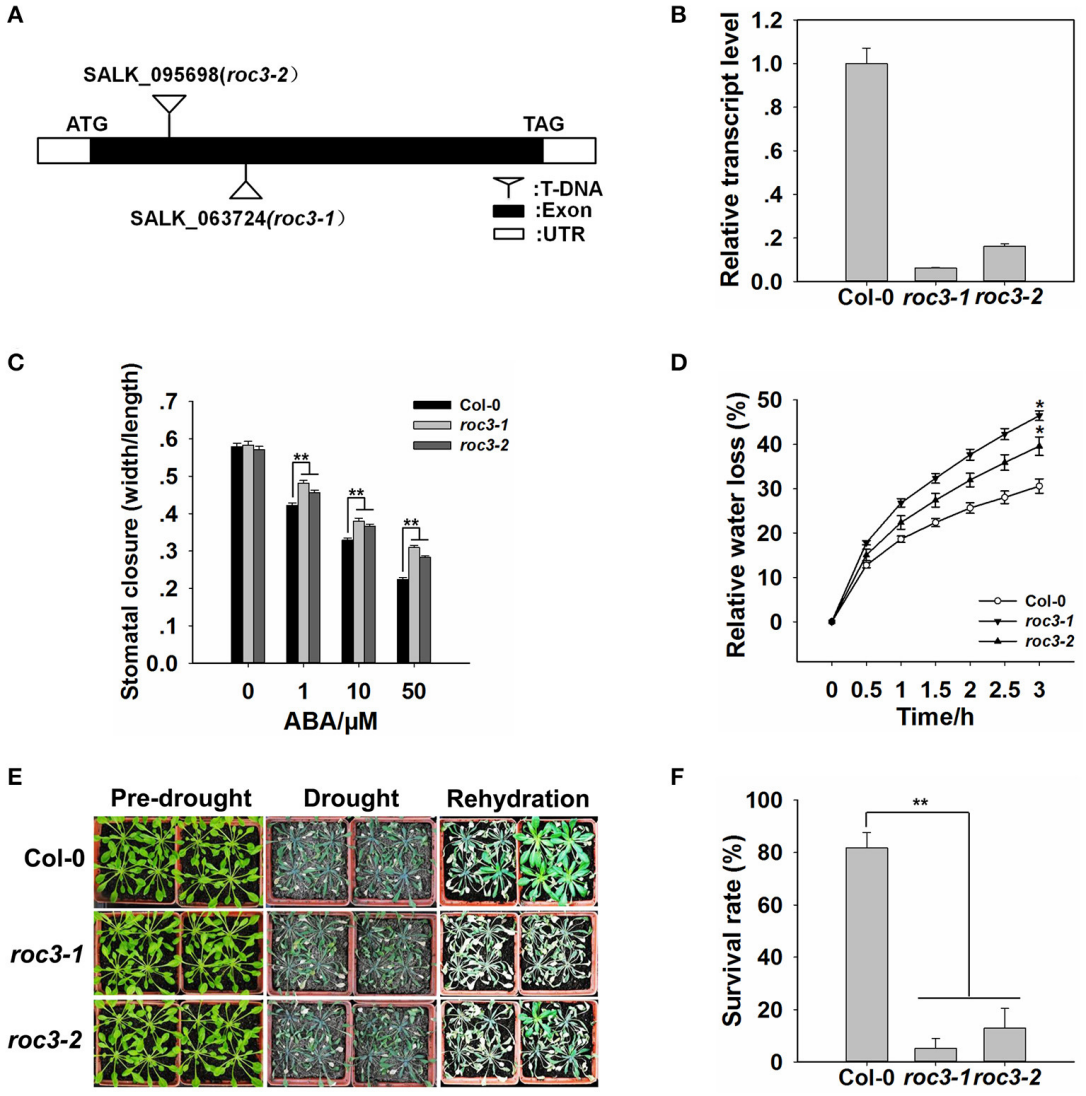
Stomatal closure in *ROC3* knock-down mutants is induced by exposure to ABA and the plants exhibit a lesser level of drought tolerance. **(A)** The T-DNA insertion points in the two independent *roc3* mutants. **(B)** Relative *ROC3* transcript abundances assessed using qRT-PCR in WT and *roc3* seedlings. Values shown in the form mean ± SE (*n* = 3). **(C)** ABA-induced stomatal closure, as quantified by the stomatal width/length ratio measured in at least 60 stomata per genotype per replicate. Error bars represent the SE (*n* = 60), **: means differed significantly from WT (*P* < 0.01). **(D)** The rate of water loss from detached rosette leaves of WT and *roc3* mutants. Values shown in the form mean ± SE (*n* = 3), *: means differed significantly from WT (*P* < 0.05). **(E)** The appearance of WT and *roc3* mutant plants grown under conditions of drought stress. **(F)** The survival rate of WT and *roc3* mutant plants grown under conditions of moisture stress. Data are shown as means ± SE (*n* = 8).

Since the expression of *ROC3* could be induced by ABA (Figure 1A) and p*ROC3::GUS* was expressed in guard cells (Figures 1H,I), we hypothesized that *ROC3* may play a role in ABA-regulated stomatal movement. Two independent T-DNA insertion *roc3* mutants (Figures 2A,B; Supplementary Figure 1) were used to examine the role of ROC3 in stomatal closure. Under control conditions, stomatal apertures did not show significant difference between either of the *roc3* mutants and WT, but when leaves were treated with a range of ABA concentrations (1–50 μM), the aperture of the mutants' stomata was clearly bigger than that of WT stomata (Figure 2C). Similarly, the rate of water loss from *roc3* mutant excised rosette leaves was higher than WT plants (Figure 2D), while the survival rate of soil-grown *roc3* mutant plants was lower than that of WT plants after rehydration (Figures 2E,F).

### The p*ROC3::ROC3* Transgene Rescues the Drought Stress Phenotype of the *roc3* Mutants

**Figure 3:**
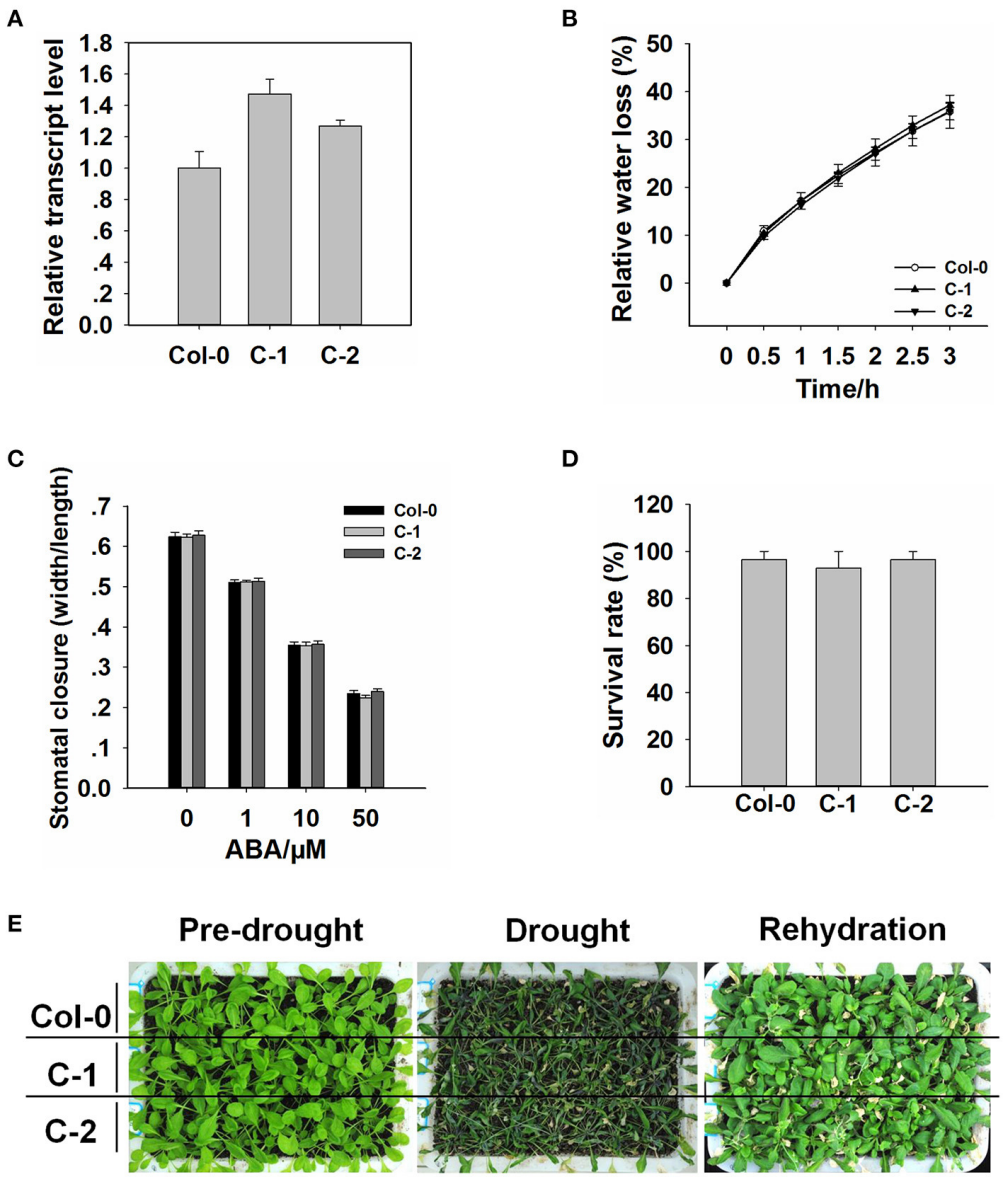
The insertion of the p*ROC3::ROC3* transgene rescues the *roc3* mutants' phenotype. **(A)** Relative *ROC3* transcript abundances assessed using qRT-PCR in WT and the complementation lines C-1 and C-2. Error bars represent the SE (*n* = 3). **(B)** The rate of water loss from rosette leaves detached from WT, C-1, and C-2 plants. Values shown in the form mean ± SE (*n* = 3). **(C)** ABA-induced stomatal closure in the WT, C-1, and C-2 leaf epidermis, stomatal aperture width/length ratios measured in at least 60 stomata per genotype per replicate. Data are shown as means ± SE (*n* = 60). **(D)** The survival rate of WT, C-1, and C-2 plants growing under drought stress conditions. Values shown in the form mean ± SE (*n* = 3). **(E)** The appearance of drought-stressed WT, C-1, and C-2 plants.

To further confirm the function of *ROC3* in stomatal regulation and drought response, we generated transgenic plants harboring p*ROC3::ROC3* in *roc3* mutant backgrounds. The abundance of *ROC3* transcript in the *ROC3* complementation lines, C1 and C2, was similar as in WT plants (Figure 3A). When C-1 and C-2 line plants were assessed either for their water loss from detached rosette leaves or their growth response to drought stress, their performance was almost indistinguishable from that of WT plants (Figures 3B,D,E). Similarly, stomatal aperture was similar between *ROC3* complementation lines and WT plants upon ABA treatment (Figure 3C).

### ROC3 Influences the Activation of S-Type Anion Channels in Plants Exposed to ABA

**Figure 4:**
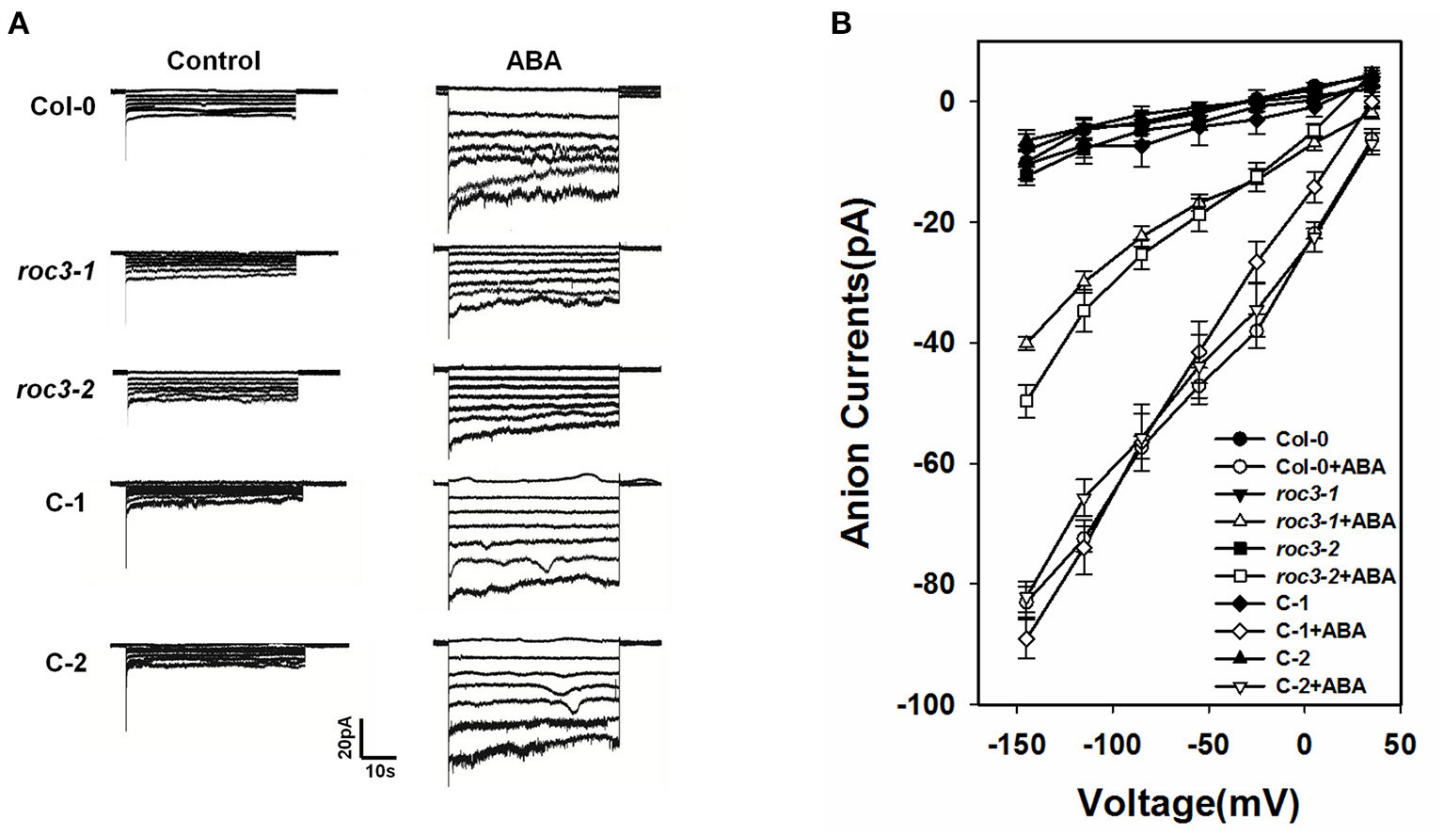
*ROC3* is involved in the ABA-induced activation of anion channels in guard cells. **(A)** Patch-clamp whole cell recordings of S-type anion currents present in guard cell protoplasts isolated from WT, *roc3* mutants and the C-1 and C-2 complementation lines, incubated in the presence/absence of 50 μM ABA. **(B)** Average current-voltage relationships of whole cell S-type anion currents. The number of guard cells measured were: WT (5), WT+ABA (6), *roc3-1* (4), *roc3-1*+ABA (6), *roc3-2* (5), *roc3-2*+ABA (6), C-1 (4), C-1+ABA (6), C-2 (4), C-2+ABA (5). Values shown in the form mean ± SE.

Previous research findings described that the activation of S-type anion channels in guard cell plasma membrane can lead to anion outflow, change in guard cell turgor, and finally promote stomatal closure (Vahisalu et al., 2008; Kim et al., 2010; Zhang et al., 2018). To investigate whether *ROC3* positively regulates ABA-induced stomatal closure through the activation of S-type anion channels, we examined the activity of S-type anion channel currents in guard cells. Under control conditions, no difference was observed for S-type anion channel currents in the guard cells of WT, *roc3* mutants and the two *ROC3* complementation lines. In contrast, in response to ABA, ABA-activated of anion currents was clearly smaller in the *roc3* mutants than in either WT or the C-1 and C-2 lines (Figures 4A,B).

### The Accumulation of Cytosolic ROS Is Reduced by the Absence of Functional ROC3

**Figure 5:**
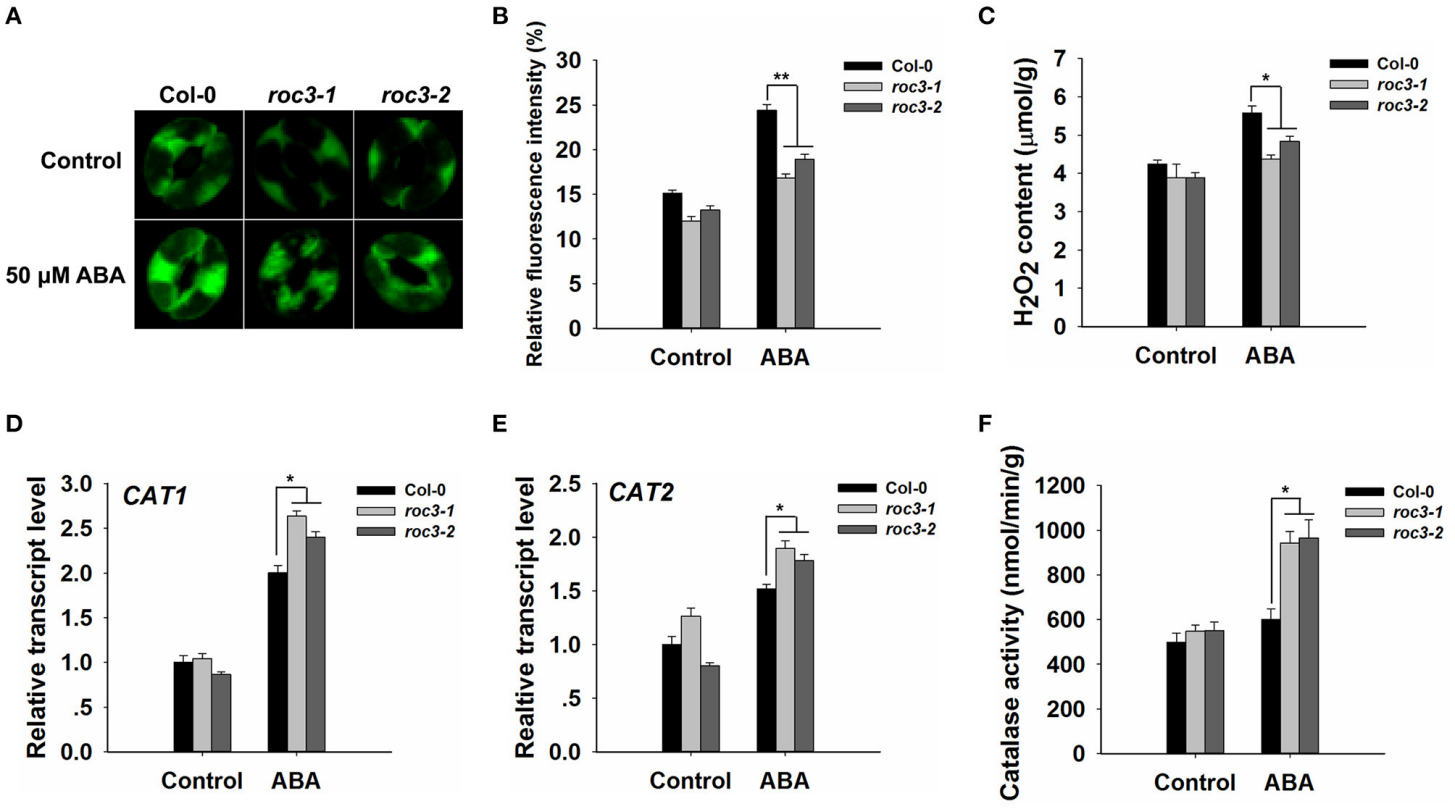
The accumulation of ROS is less efficient in *roc3* mutants than in WT guard cells. **(A)** Laser confocal micrographs revealing the ROS content of guard cells sampled from WT or *roc3* mutant plants either exposed or not exposed to ABA; the fluorescent signal is generated from CM-H_2_DCFDA. **(B)** Quantification of the fluorescence intensities shown in **(A)**. At least 100 guard cells were sampled from each genotype. Error bars represent the SE (*n* = 100). The experiments were repeated three times with similar results. **(C)** The H_2_O_2_ content in the leaf of WT and *roc3* plants, either exposed or not exposed to 50 μM ABA. Values shown in the form mean ± SE (*n* = 3). **(D,E)** Relative transcript abundances assessed using qRT-PCR in plants either exposed or not exposed to ABA: **(D)** *CAT1*, **(E)** *CAT2*. Data are shown as means ± SE (*n* = 3). **(F)** Quantification of catalase activity assay in WT and *roc3* mutants, either exposed or not exposed to 50 μM ABA. Values shown in the form mean ± SE (*n* = 3). *,**: values differed significantly from WT (*P* < 0.05, 0.01).

ROS are crucial messenger molecules that participate in ABA regulation of anion channels and stomatal movement (Pei et al., 2000; Zhang et al., 2001; Munemasa et al., 2007). We wanted to know whether *ROC3* plays any regulatory roles in ABA-triggered ROS accumulation. Firstly, a fluorescence-based assay was used to measure the ROS content of WT and *roc3* guard cells. In the control condition, the guard cells of *roc3* showed reduced accumulation of ROS compared to WT. In response to ABA, the guard cells of WT showed higher accumulation of ROS compared to the guard cells of *roc3* (Figures 5A,B; Supplementary Figure 2). Similarly, H_2_O_2_ levels were lower in ABA-treated *roc3* than in ABA-treated WT plants (Figure 5C). After ABA treatment, the absence of *ROC3* had no obvious effect on the transcripts of two NADPH oxidase genes, *RbohD* and *RbohF*, which are involved in the ABA induced ROS production (Supplementary Figure 3). However, there was a moderate effect on the expression of both *CAT1* and *CAT2*, genes encode catalases which act as ROS scavengers: the expression of both genes was greater in *roc3* mutants than in WT plants upon ABA treatment (Figures 5D,E). Similarly, leaf catalase activity was higher in the mutants' leaves than WT in response to ABA treatment (Figure 5F).

### ROS Accumulation Participates in *ROC3*-Regulated Stomatal Closure

**Figure 6:**
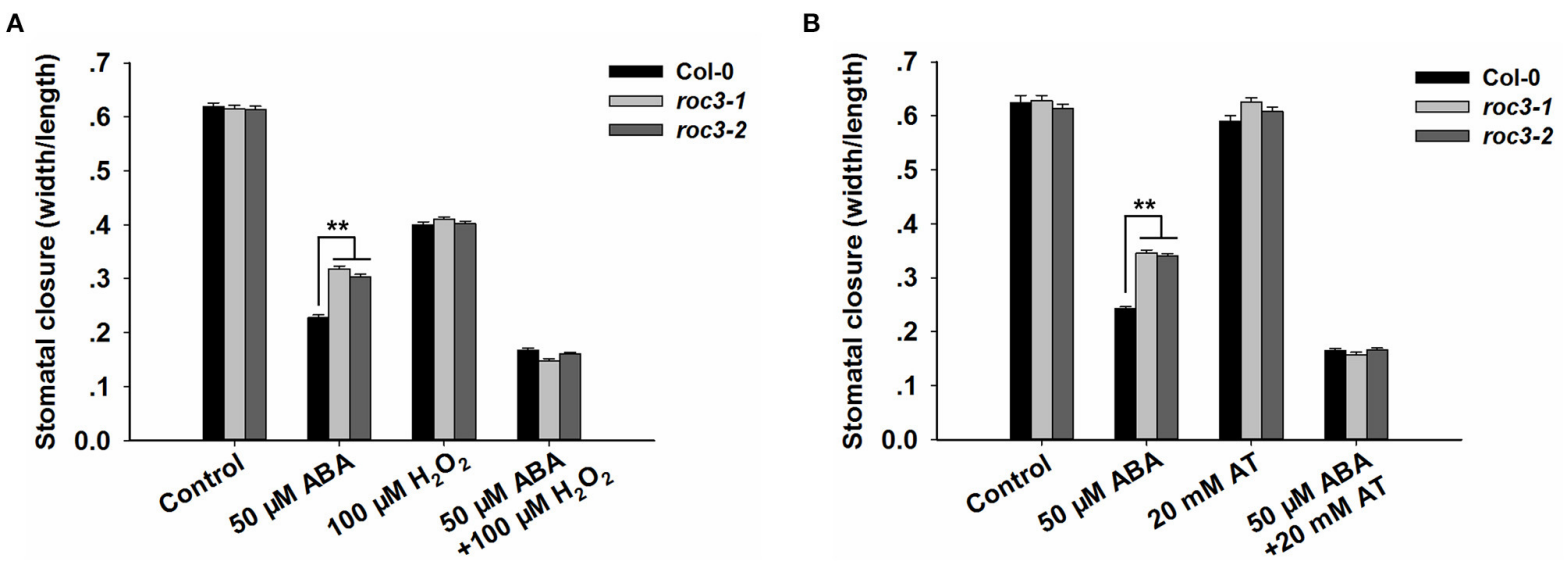
ROC3 participates in ROS signal-regulated stomatal closure. **(A)** Stomatal closure assays of WT and *roc3* mutant leaf epidermis treated with either ABA and/or H_2_O_2_, and stomatal aperture width/length ratios measured in at least 60 stomata per genotype per replicate. Means shown in the form mean ± SE (*n* = 60), **: means differed significantly from WT (*P* < 0.01). **(B)** Stomatal closure assays of WT and *roc3* mutant leaf epidermis treated with ABA and/or AT, and stomatal aperture width/length ratios measured in at least 60 stomata per genotype per replicate. Values shown in the form mean ± SE (*n* = 60), **: means differed significantly from WT (P < 0.01). Both of the experiments were repeated three times.

To explore whether *ROC3* was involved in ABA-induced stomatal closure by affecting ROS accumulation, we carried out stomatal closure experiments with H_2_O_2_ or AT (an inhibitor of catalase) treatment, in the presence or absence of ABA. The absence of functional ROC3 had no discernible effect on H_2_O_2_-induced stomatal closure in leaf epidermis strips, but the genotypic difference in stomatal aperture induced by ABA treatment was abolished by addition of exogenous H_2_O_2_ (Figure 6A). AT treatment had little influence on the stomatal movement, but the presence of AT promoted ABA-induced stomatal closure and abolished the difference in ABA-promoted stomatal closure between WT and *roc3* (Figure 6B).

### ROC3 Influences the Expression Levels of Stress-Responsive Genes

**Figure 7:**
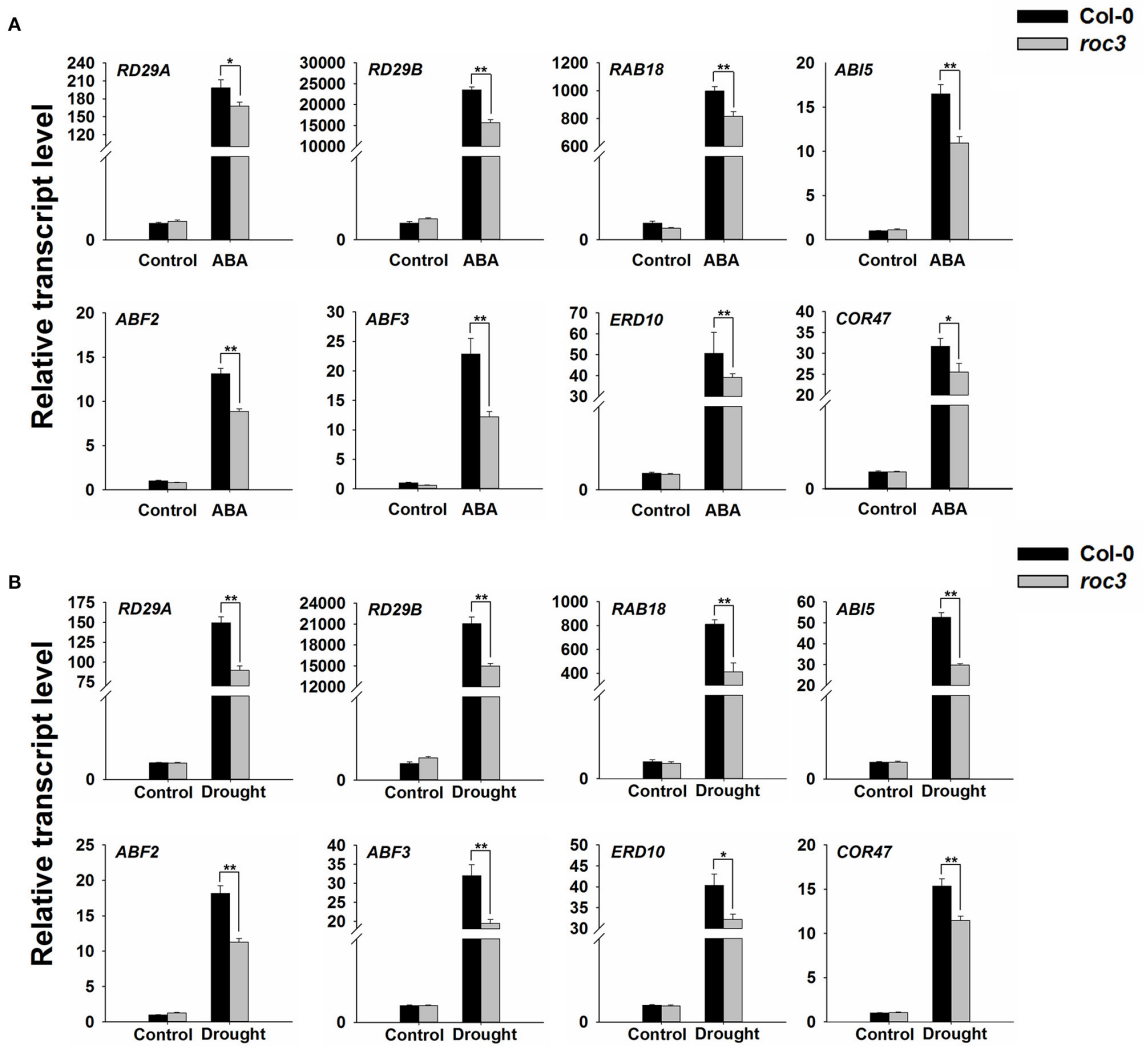
Transcriptional profiling of the indicated genes associated with the stress response in *roc3* mutant exposed to either ABA treatment or drought stress. Relative transcript abundances assessed using qRT-PCR in plants subjected to **(A)** 6 h exposure to ABA, **(B)** 6 h exposure to drought stress. Means shown in the form mean ± SE (*n* = 3), *, **: means differed significantly from control treatment (*P* < 0.05, 0.01).

Previous findings have shown that, ABA can induce the expression of many downstream key genes involved in plant dehydration stress (Yamaguchi-shinozaki and Shinozaki, 2006; Ju et al., 2020). An examination of the effect of ROC3 on the transcription of a series of genes known to participate in the stress response (*RD29A, RD29B, RAB18, ABI5, ABF2, ABF3, ERD10*, and *COR47*) showed that in each gene, transcript abundances were lower in *roc3* mutant than in WT plants in response to ABA treatment or drought stress (Figure 7).

## Discussion

Drought exerts significant negative effects on crop productivity (Boyer, 1982; Venuprasad et al., 2007). Attempts to increase the resilience of crop varieties to this stress via conventional breeding has achieved only a modest level of success, while the potential of transgenic technology to address this issue has also been demonstrated (Manavalan et al., 2009; Hu and Xiong, 2014). The key to the success of a transgenic-based improvement strategy for a specific stress like drought is very much dependent on the specific choice of transgene(s). Therefore, the identification of genes contributing to a plant's defense against drought remains a major research priority and it is very much necessary to understand how the candidate genes affect the plant's response to the stress. Most of the water lost by plants passes through the stomata, so control on stomatal movement will have a major impact on the plant's hydration status (Schroeder et al., 2001; Martin-StPaul et al., 2017; Agurla et al., 2018). It has been established that ABA-triggered activation of guard cell anion channels results in the efflux of anions, which in turn reduces the turgor of guard cells to close the stomata (Cutler et al., 2010; Hubbard et al., 2010; Lee and Luan, 2012) and other proteins also have been implicated in this process.

The outcome of our experiments support the notion that the CYP member ROC3 of *A. thaliana* acts as a positive regulator of ABA-induced stomatal closure. *ROC3* was found to be induced by both ABA treatment and the drought stress (Figures 1A,B). p*ROC3::GUS* activity was detected in seedling, leaf, flower, silique, and was mildly induced in guard cells upon ABA treatment (Figures 1C–I), implying that *ROC3* may play a role in guard cell ABA signaling. The stomata of *roc3* mutant plants were more open than those of WT plants in ABA treatment (Figure 2C), and the mutants were also less tolerant of drought (Figures 2D–F). Supportively, *ROC3* complementation lines, C1 and C2, restored the WT phenotype (Figure 3), suggesting that ROC3 plays a positive regulatory role in ABA-induced stomatal closure and the drought stress response. Plasma membrane localized anion channels are activated by the accumulation of ABA in guard cells, leading to the depolarization of plasma membrane and anion outflow which causes the reduction of guard cell turgor, that ultimately leads to stomatal closure (MacRobbie, 1998; Hedrich, 2012; Roelfsema et al., 2012; Hedrich and Geiger, 2017). A patch clamp experiment demonstrated that in protoplasts exposed to ABA, S-type anion channel activity was lower in *roc3* mutants compared to WT or the *ROC3* complementation line plants (Figure 4), indicating that the absence of functional ROC3 causes reduced outflow of anions from the guard cells in response to ABA, hence stomatal aperture remains larger. It remains to be explored how ROC3 influences anion channel activity in response to drought stress or ABA treatment. Further research will be conducted to analyze whether *ROC3* can regulate the expression of the known genes encoding anion channels (e.g., *SLAC1*) or these channels activity regulation (e.g., *CPK3*).

It is well-known that ABA signaling in guard cells requires the participation of multiple signaling elements. Among them, ROS (notably H_2_O_2_) work as crucial secondary messengers in regulating ABA-induced stomatal closure (Hua et al., 2012; Gayatri et al., 2013; Song et al., 2014; Dietz et al., 2016; Li et al., 2017). A rise in leaf ABA content, due to endogenous processes or an exogenous ABA treatment, stimulates the production of ROS (Bright et al., 2006; Kreslavski et al., 2012). The concentration of ROS in the leaves of *roc3* mutant plants exposed to ABA was less than in WT leaves (Figures 5A–C; Supplementary Figure 2), consistent with the proposed positive regulatory roles of ROC3 in both stomatal closure and the drought stress response. Interestingly, the stomatal aperture of *roc3* was similar to Col-0's when simultaneously exposed to ABA and H_2_O_2_, and the hyposensitive phenotype of the *roc3* mutants with respect to ABA-induced stomatal closure was abolished when supplemented with exogenous H_2_O_2_ (Figure 6A). It is possible that ROS act as downstream components during ROC3-modulated stomatal closure. Previously it has been shown that the ABA-induced production of ROS is primarily catalyzed by plasma membrane localized NADPH oxidases, while ROS neutralization is carried out by various enzymatic (notably catalase) and non-enzymatic antioxidants (Willekens et al., 1995; Zhang et al., 2001; Dietz, 2003; Kwak et al., 2003; Mittler et al., 2004; Mhamdi et al., 2010). In response to ABA, *roc3* mutants showed the upregulation of both *CAT1* and *CAT2* compared to WT plants (Figures 5D,E), although the *roc3* plants did not show any differences in the gene expression profile of either *RbohD* or *RbohF* (Supplementary Figure 3). Furthermore, higher catalase activity was observed in *roc3* mutants than in WT in response to ABA (Figure 5F). These findings suggested that reduced accumulation of ROS in *roc3* mutants was due to a higher level of catalase activity. This hypothesis was tested by stomatal movement experiments with a known catalase inhibitor, AT. The difference of stomatal aperture between WT and *roc3* mutants in ABA treatment was abolished when AT was combined with ABA (Figure 6B). Our findings propose that by unknown mechanism ROC3 suppresses catalase activity, thereby affecting the accumulation of ROS that in turn positively regulates ABA-induced stomatal closure.

Many genes involved in the drought stress response are known to be induced by ABA treatment (Kang et al., 2002; Yamaguchi-shinozaki and Shinozaki, 2006; Kovacs et al., 2008; Liu and Stone, 2010; Ma et al., 2019; Ju et al., 2020). Comparisons of the profiles of the expression of some of these genes between *roc3* and WT leaves which were either treated with ABA or subjected to drought stress indicated that their expression in response to either stress was somewhat compromised in the mutant (Figure 7), providing an additional explanation for the reduced sensitivity of *roc3* to exogenous ABA or drought stress. The mechanistic basis of this interaction is unknown.

**Figure 8:**
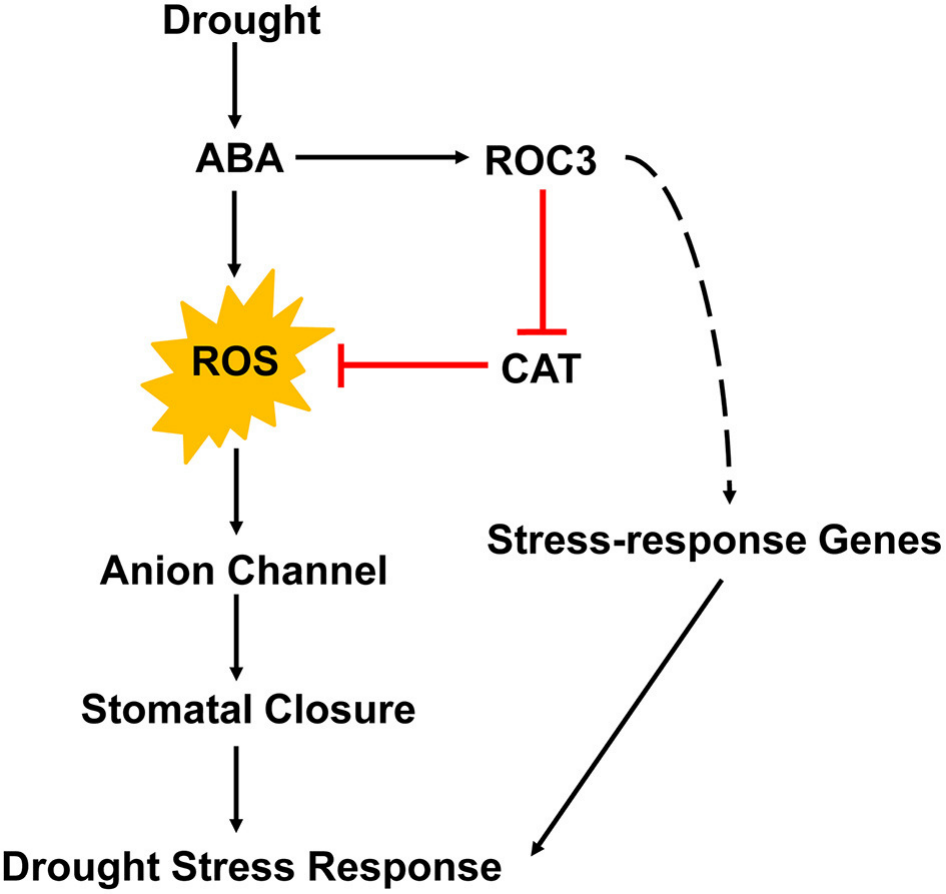
A proposed working model for the role of ROC3 in the regulation of stomatal movement and the drought stress response of *A. thaliana*.

A working model summarizing the participation of ROC3 in ABA-induced stomatal closure and the drought stress response of *A. thaliana* is depicted in Figure 8. Drought stress promotes the accumulation of ABA in guard cells. Then, ABA induces the production of ROS and subsequently the activation of anion channels that leads to stomatal closure, thereby cutting off the major route by which water is lost from the plant. Our current findings suggest that ROC3 acts as a positive regulator in ABA-induced stomatal closure. ROC3 inhibits CAT activity, thereby maintaining a sufficient level of ROS in guard cells to ensure stomatal closure. At the same time, ROC3 also contributes to both ABA signaling and tolerance to drought stress by maintaining the expression of a number of genes that play important roles in plant's stress response. The present experimental findings have revealed a novel function for ROC3 beyond its known involvement in protein folding, the catalysis of *cis-trans* isomerization of proline imidic peptide bonds in oligopeptides and the response to pathogen infection. The exact mechanism remains to be elucidated how ROC3 interacts with ABA signaling machineries, how it suppresses catalase activity, and how it influences the expression of stress response genes.

## Data Availability Statement

The raw data supporting the conclusions of this article will be made available by the authors, without undue reservation.

## Author Contributions

WZ designed the experiments, interpreted the results, and edited the manuscript. HL performed the major experiments, analyzed the results, and wrote the manuscript. CY and DL performed a few experiments. JS, BA, MW, and DC designed a few experiments and analyzed some data. All authors contributed to the article and approved the submitted version.

## Conflict of Interest

The authors declare that the research was conducted in the absence of any commercial or financial relationships that could be construed as a potential conflict of interest.

## Supplementary Material

Supplementary Table 1 Primer sequences used in the experiments.

Supplementary Figure 1 PCR screening of homozygous T-DNA mutants of *ROC3*. 1: *roc3-1*-LP+LBb1.3; 2: *roc3-1*-RP+LBb1.3; 3: *roc3-1*-LP+ *roc3-1*-RP; 4: *roc3-2*-LP+LBb1.3; 5: *roc3-2*-RP+LBb1.3; 6: *roc3-2*-LP+ *roc3-2*-RP; M: Marker.

Supplementary Figure 2 Fluorescence micrographs revealing the ROS content of guard cells sampled from WT or *roc3* mutant plants either exposed or not exposed to ABA; the fluorescent signal is generated from CM-H_2_DCFDA and captured by a conventional fluorescence microscope. There are 60 guard cells were sampled from each genotype. Error bars represent the SE (*n* = 60), ^**^: means differed significantly from Col-0 (*P* <0.01).

Supplementary Figure 3 Transcriptional profiling of two genes encoding NADPH/respiratory burst oxidase proteins. Relative transcript abundances of **(A)** *RBOHD*, **(B)** *RBOHF*, assessed using qRT-PCR, in WT or *roc3* mutants either exposed or not exposed to ABA. Values shown in the form mean ± SE (*n* = 3).

## References

[B1] Acharya B. R. Jeon B. W. Zhang W. Assmann S. M. (2013). Open Stomata 1 (OST1) is limiting in abscisic acid responses of Arabidopsis guard cells. New phytol. 200, 1049–1063. 10.1111/nph.12469 24033256

[B2] Agurla S. Gahir S. Munemasa S. Murata Y. Raqhavendra A. S. (2018). Mechanism of stomatal closure in plants exposed to drought and cold stress. Adv. Exp. Med. Biol. 1081, 215–232. 10.1007/978-981-13-1244-1_12 30288712

[B3] Apel K. Hirt H. (2004). Reactive oxygen species: metabolism, oxidative stress, and signal transduction. Annu. Rev. Plant Biol. 55, 373–399. 10.1146/annurev.arplant.55.031903.141701 15377225

[B4] Boyer J. S. (1982). Plant productivity and environment. Science 218, 443–448. 10.1126/science.218.4571.443 17808529

[B5] Bright J. Desikan R. Hancock J. T. Weir I. S. Neill S. J. (2006). ABA-induced NO generation and stomatal closure in *Arabidopsis* are dependent on H_2_O_2_ synthesis. Plant J. 45, 113–122. 10.1111/j.1365-313X.2005.02615.x 16367958

[B6] Clough S. J. Bent A. F. (1998). Floral dip: a simplified method for Agrobacterium-mediated transformation of *Arabidopsis thaliana*. Plant J. 16, 735–743. 10.1046/j.1365-313x.1998.00343.x 10069079

[B7] Corpas F. J. Barroso J. B. del Río L. A. (2001). Peroxisomes as a source of reactive oxygen species and nitric oxide signal molecules in plant cells. Trends Plant Sci. 6, 145–150. 10.1016/S1360-1385(01)01898-2 11286918

[B8] Cutler S. R. Rodriguez P. L. Finkelstein R. R. Abrams S. R. (2010). Abscisic acid: emergence of a core signaling network. Annu. Rev. Plant Biol. 61, 651–679. 10.1146/annurev-arplant-042809-112122 20192755

[B9] Dietz K. J. (2003). Plant peroxiredoxins. Annu. Rev. Plant Biol. 54, 93–107. 10.1146/annurev.arplant.54.031902.134934 14502986

[B10] Dietz K. J. Mittler R. Noctor G. (2016). Recent progress in understanding the role of reactive oxygen species in plant cell signaling. Plant Physiol. 171, 1535–1539. 10.1104/pp.16.00938 27385820 PMC4936595

[B11] Gayatri G. Agurla S. Raghavendra A. S. (2013). Nitric oxide in guard cells as an important secondary messenger during stomatal closure. Front. Plant Sci. 4:425. 10.3389/fpls.2013.00425 24194741 PMC3810675

[B12] Handschumacher R. E. Harding M. W. Rice J. Drugge R. J. Speicher D. W. (1984). Cyclophilin: a specific cytosolic binding protein for cyclosporin A. Science 226, 544–546. 10.1126/science.6238408 6238408

[B13] He Z. Li L. Luan S. (2004). Immunophilins and parvulins. Superfamily of peptidyl prolyl isomerases in Arabidopsis. Plant Physiol. 134, 1248–1267. 10.1104/pp.103.031005 15047905 PMC419802

[B14] Hedrich R. (2012). Ion channels in plants. Physiol. Rev. 92, 1777–1811. 10.1152/physrev.00038.2011 23073631

[B15] Hedrich R. Geiger D. (2017). Biology of SLAC1-type anion channels-from nutrient uptake to stomatal closure. New Phytol. 216, 46–61. 10.1111/nph.14685 28722226

[B16] Hu H. Xiong L. (2014). Genetic engineering and breeding of drought-resistant crops. Annu. Rev. Plant Biol. 65, 715–741. 10.1146/annurev-arplant-050213-040000 24313844

[B17] Hua D. P. Wang C. He J. N. Liao H. Duan Y. Zhu Z. Q.. (2012). A plasma membrane receptor kinase, GHR1, mediates abscisic acid, and hydrogen peroxide-regulated stomatal movement in *Arabidopsis*. Plant Cell. 24, 2546–2561. 10.1105/tpc.112.100107 22730405 PMC3406912

[B18] Hubbard K. E. Nishimura N. Hitomi K. Getzoff E. D. Schroeder J. I. (2010). Early abscisic acid signal transduction mechanisms: newly discovered components and newly emerging questions. Genes Dev. 24, 1695–1708. 10.1101/gad.1953910 20713515 PMC2922499

[B19] Jannat R. Uraji M. Morofuji M. Islam M. M. Bloom R. E. Nakamura Y.. (2011). Roles of intracellular hydrogen peroxide accumulation in abscisic acid signaling in *Arabidopsis* guard cells. J. Plant Physiol. 168, 1919–1926. 10.1016/j.jplph.2011.05.006 21665322 PMC4073789

[B20] Ju Y. L. Min Z. Yue X. F. Zhang Y. L. Zhang J. X. Zhang Z. Q.. (2020). Overexpression of grapevine VvNAC08 enhances drought tolerance in transgenic Arabidopsis. Plant Physiol. Biochem. 151, 214–222. 10.1016/j.plaphy.2020.03.028 32229406

[B21] Kang J. Y. Choi H. I. Im M. Y. Kim S. Y. (2002). Arabidopsis basic leucine zipper proteins that mediate stress-responsive abscisic acid signaling. Plant Cell 14, 343–357. 10.1105/tpc.010362 11884679 PMC152917

[B22] Kim S. K. You Y. N. Park J. C. Joung Y. Kim B. G. Ahn J. C.. (2012). The rice thylakoid lumenal cyclophilin OsCYP20-2 confers enhanced environmental stress tolerance in tobacco and *Arabidopsis*. Plant Cell Rep. 31, 417–426. 10.1007/s00299-011-1176-x 22041789

[B23] Kim T. H. Böhmer M. Hu H. Nishimura N. Schroeder J. I. (2010). Guard cell signal transduction network: advances in understanding abscisic acid, CO_2_, and Ca^2+^ signaling. Annu. Rev. Plant Biol. 61, 561–591. 10.1146/annurev-arplant-042809-112226 20192751 PMC3056615

[B24] Kollist H. Nuhkat M. Roelfsema M. R. (2014). Closing gaps: linking elements that control stomatal movement. New phytol. 203, 44–62. 10.1111/nph.12832 24800691

[B25] Kovacs D. Kalmar E. Torok Z. Tompa P. (2008). Chaperone activity of ERD10 and ERD14, two disordered stress-related plant proteins. Plant Physiol. 147, 381–390. 10.1104/pp.108.118208 18359842 PMC2330285

[B26] Kreslavski V. D. Los D. A. Allakhverdiev S. I. Kuznetsov V. V. (2012). Signaling role of reactive oxygen species in plants under stress. Russ. J. Plant Physl. 59, 141–154. 10.1134/S1021443712020057

[B27] Kwak J. M. Mori I. C. Pei Z. M. Leonhardt N. Torres M. A. Dangl J. L.. (2003). NADPH oxidase *AtrbohD* and *AtrbohF* genes function in ROS-dependent ABA signaling in *Arabidopsis*. EMBO J. 22, 2623–2633. 10.1093/emboj/cdg277 12773379 PMC156772

[B28] Langridge P. Reynolds M. P. (2015). Genomic tools to assist breeding for drought tolerance. Curr. Opin. Biotechnol. 32, 130–135. 10.1016/j.copbio.2014.11.027 25531270

[B29] Lee S. C. Luan S. (2012). ABA signal transduction at the crossroad of biotic and abiotic stress responses. Plant Cell Environ. 35, 53–60. 10.1111/j.1365-3040.2011.02426.x 21923759

[B30] Lee S. S. Park H. J. Yoon D. H. Kim B. G. Ahn J. C. Luan S.. (2015). Rice cyclophilin OsCYP18-2 is translocated to the nucleus by an interaction with SKIP and enhances drought tolerance in rice and *Arabidopsis*. Plant Cell Environ. 38, 2071–2087. 10.1111/pce.12531 25847193

[B31] Li C. L. Wang M. Wu X. M. Chen D. H. Lv H. J. Shen J. L.. (2016). THI1, a thiamine thiazole synthase, interacts with Ca^2+^-dependent protein kinase CPK33 and modulates the S-type anion channels and stomatal closure in *Arabidopsis*. Plant Physiol. 170, 1090–1104. 10.1104/pp.15.01649 26662273 PMC4734576

[B32] Li J. Wang X. Q. Watson M. B. Assmann S. M. (2000). Regulation of abscisic acid-induced stomatal closure and anion channels by guard cell AAPK kinase. Science 287, 300–303. 10.1126/science.287.5451.300 10634783

[B33] Li J. J. Li Y. Yin Z. G. Jiang J. H. Zhang M. H. Guo X.. (2017). OsASR5 enhances drought tolerance through a stomatal closure pathway associated with ABA and H_2_O_2_ signalling in rice. Plant Biotechnol. J. 15, 183–196. 10.1111/pbi.12601 27420922 PMC5258865

[B34] Lin H. Wang R. Qian Q. Yan M. Meng X. Fu Z.. (2009). DWARF27, an iron-containing protein required for the biosynthesis of strigolactones, regulates rice tiller bud outgrowth. Plant Cell 21, 1512–1525. 10.1105/tpc.109.065987 19470589 PMC2700539

[B35] Liu H. Stone S. L. (2010). Abscisic acid increases *Arabidopsis* ABI5 transcription factor levels by promoting KEG E3 ligase self-ubiquitination and proteasomal degradation. Plant Cell 22, 2630–2641. 10.1105/tpc.110.076075 20682837 PMC2947163

[B36] Ma Y. Cao J. Chen Q. He J. Liu Z.. (2019). The kinase CIPK11 functions as a negative regulator in drought stress response in *Arabidopsis*. Int. J. Mol. Sci. 20:2422. 10.3390/ijms20102422 31100788 PMC6566343

[B37] MacRobbie E. A. (1998). Signal transduction and ion channels in guard cells. Philos. Trans. R. Soc. Lond. B Biol. Sci. 353, 1475–1488. 10.1098/rstb.1998.0303 9800209 PMC1692354

[B38] Manavalan L. P. Guttikonda S. K. Tran L. S. Nguyen H. T. (2009). Physiological and molecular approaches to improve drought resistance in soybean. Plant Cell Physiol. 50, 1260–1276. 10.1093/pcp/pcp082 19546148

[B39] Martin-StPaul N. Delzon S. Cochard H. (2017). Plant resistance to drought depends on timely stomatal closure. Ecol. Lett. 20, 1437–1447. 10.1111/ele.12851 28922708

[B40] Mhamdi A. Queval G. Chaouch S. Vanderauwera S. Van Breusegem F. Noctor G. (2010). Catalase function in plants: a focus on Arabidopsis mutants as stress-mimic models. J. Exp. Bot. 61, 4197–4220. 10.1093/jxb/erq282 20876333

[B41] Miao Y. Lv D. Wang P. Wang X. C. Chen J. Miao C.. (2006). An *Arabidopsis* glutathione peroxidase functions as both a redox transducer and a scavenger in abscisic acid and drought stress responses. Plant Cell 18, 2749–2766. 10.1105/tpc.106.044230 16998070 PMC1626619

[B42] Mittler R. (2002). Oxidative stress, antioxidants, and stress tolerance. Trends Plant Sci. 7, 405–410. 10.1016/S1360-1385(02)02312-9 12234732

[B43] Mittler R. Vanderauwera S. Gollery M. Van Breusegem F. (2004). Reactive oxygen gene network of plants. Trends Plant Sci. 9, 490–498. 10.1016/j.tplants.2004.08.009 15465684

[B44] Mori I. C. Murata Y. Yang Y. Munemasa S. Wang Y. F. Andreoli S.. (2006). CDPKs CPK6 and CPK3 function in ABA regulation of guard cell S-type anion- and Ca^2+^- permeable channels and stomatal closure. PLoS Biol. 4:e327. 10.1371/journal.pbio.0040327 17032064 PMC1592316

[B45] Munemasa S. Hauser F. Park J. Waadt R. Brandt B. Schroeder J. I. (2015). Mechanisms of abscisic acid-mediated control of stomatal aperture. Curr. Opin. Plant Biol. 28, 154–162. 10.1016/j.pbi.2015.10.010 26599955 PMC4679528

[B46] Munemasa S. Oda K. Watanabe-Sugimoto M. Nakamura Y. Shimoishi Y. Murata Y. (2007). The coronatine-insensitive 1 mutation reveals the hormonal signaling interaction between abscisic acid and methyl jasmonate in Arabidopsis guard cells. Specific impairment of ion channel activation and second messenger production. Plant Physiol. 143, 1398–1407. 10.1104/pp.106.091298 17220365 PMC1820907

[B47] Murashige T. Skoog F. (1962). A revised medium for rapid growth and bioassays with tobacco tissue cultures. Physiol. Plant. 15, 473–472. 10.1111/j.1399-3054.1962.tb08052.x

[B48] Murata Y. Mori I. C. Munemasa S. (2015). Diverse stomatal signaling and the signal integration mechanism. Annu. Rev. Plant Biol. 66, 369–392. 10.1146/annurev-arplant-043014-114707 25665132

[B49] Nyathi Y. Baker A. (2006). Plant peroxisomes as a source of signalling molecules. Biochem. Biophys. Acta 1763, 1478–1495. 10.1016/j.bbamcr.2006.08.031 17030442

[B50] Osakabe Y. Osakabe K. Shinozaki K. Tran L. S. (2014). Response of plants to water stress. Front. Plant Sci. 5:86. 10.3389/fpls.2014.00086 24659993 PMC3952189

[B51] Palma J. M. Corpas F. J. del Río L. A. (2009). Proteome of plant peroxisomes: new perspectives on the role of these organelles in cell biology. Proteomics 9, 2301–2312. 10.1002/pmic.200700732 19343723

[B52] Pei Z. M. Kuchitsu K. Ward J. M. Schwarz M. Schroeder J. I. (1997). Differential abscisic acid regulation of guard cell slow anion channels in *Arabidopsis* wild-type and abi1 and abi2 mutants. Plant Cell 9, 409–423. 10.1105/tpc.9.3.409 9090884 PMC156927

[B53] Pei Z. M. Murata Y. Benning G. Thomine S. Klüsener B. Allen G. J.. (2000). Calcium channels activated by hydrogen peroxide mediate abscisic acid signalling in guard cells. Nature 406, 731–734. 10.1038/35021067 10963598

[B54] Pogorelko G. V. Mokryakova M. Fursova O. V. Abdeeva I. Piruzian E. S. Bruskin S. A. (2014). Characterization of three *Arabidopsis thaliana* immunophilin genes involved in the plant defense response against *Pseudomonas syringae*. Gene 538, 12–22. 10.1016/j.gene.2014.01.029 24440291

[B55] Raghavendra A. S. Gonugunta V. K. Christmann A. Grill E. (2010). ABA perception and signaling. Trends Plant Sci. 15, 395–401. 10.1016/j.tplants.2010.04.006 20493758

[B56] Roelfsema M. R. Hedrich R. Geiger D. (2012). Anion channels: master switches of stress responses. Trends Plant Sci. 17, 221–229. 10.1016/j.tplants.2012.01.009 22381565

[B57] Santos I. B. D. Park S. W. (2019). Versatility of cyclophilins in plant growth and survival: a case study in *Arabidopsis*. Biomolecules 9:20. 10.3390/biom9010020 30634678 PMC6358970

[B58] Schroeder J. I. Allen G. J. Hugouvieux V. Kwak J. M. Waner D. (2001). Guard cell signal transdution. Annu. Rev. Plant Physiol. Plant Mol. Biol. 52, 627–658. 10.1146/annurev.arplant.52.1.627 11337411

[B59] Schroeder J. I. Hagiwara S. (1989). Cytosolic calcium regulates ion channels in the plasma membrane of Vicia faba guard cells. Nature 338, 427–430. 10.1038/338427a08952952

[B60] Seo M. Koshiba T. (2011). Transport of ABA from the site of biosynthesis to the site of action. J. Plant Res. 124, 501–507. 10.1007/s10265-011-0411-4 21416315

[B61] Sheen J. (2001). Signal transduction in maize and Arabidopsis mesophyll protoplasts. Plant Physiol. 127, 1466–1475. 10.1104/pp.010820 11743090 PMC1540179

[B62] Song Y. Miao Y. Song C. P. (2014). Behind the scenes: the roles of reactive oxygen species in guard cells. New Phytol. 202, 1121–1140. 10.1111/nph.12565 24188383

[B63] Vahisalu T. Kollist H. Wang Y. F. Nishimura N. Chan W. Y. Valerio G.. (2008). SLAC1 is required for plant guard cell S-type anion channel function in stomatal signalling. Nature 452, 487–491. 10.1038/nature06608 18305484 PMC2858982

[B64] Venuprasad R. Lafitte H. R. Atlin G. N. (2007). Response to direct selection for grain yield under drought stress in rice. Crop Sci. 47, 285–293. 10.2135/cropsci2006.03.0181

[B65] Wang X. Shi X. Hao B. Ge S. Luo J. (2005). Duplication and DNA segmental loss in the rice genome: implications for diploidization. New Phytol. 165, 937–946. 10.1111/j.1469-8137.2004.01293.x 15720704

[B66] Wang X. Q. Ullah H. Jones A. M. Assmann S. M. (2001). G protein regulation of ion channels and abscisic acid signaling in *Arabidopsis* guard cells. Science 292, 2070–2072. 10.1126/science.1059046 11408655

[B67] Willekens H. Chamnongpol S. Davey M. Schraudner M. Langebartels C. Montagu M. V.. (1997). Catalase is a sink for H_2_O_2_ and is indispensable for stress defence in C_3_ plants. EMBO J. 16, 4806–4816. 10.1093/emboj/16.16.4806 9305623 PMC1170116

[B68] Willekens H. Inzé D. Van Montagu M. Van Camp W. (1995). Catalases in plants. Mol. Breed. 1, 207–228. 10.1007/BF02277422

[B69] Xiao C. Chen F. Yu X. Lin C. Fu Y. F. (2009). Over-expression of an AT-hook gene, AHL22, delays flowering, and inhibits the elongation of the hypocotyl in *Arabidopsis thaliana*. Plant Mol. Biol. 71, 39–50. 10.1007/s11103-009-9507-9 19517252

[B70] Yamaguchi-shinozaki K. Shinozaki K. (2006). Transcriptional regulatory networks in cellular responses and tolerance to dehydration and cold stresses. Annu. Rev. Plant Biol. 57, 781–803. 10.1146/annurev.arplant.57.032905.105444 16669782

[B71] Yoon D. H. Lee S. S. Park H. J. Lyu J. I. Chong W. S. Liu J. R.. (2016). Overexpression of OsCYP19-4 increases tolerance to cold stress and enhances grain yield in rice (*Oryza sativa*). J. Exp. Bot. 67, 69–82. 10.1093/jxb/erv421 26453745 PMC4682425

[B72] Zhang J. Wang N. Miao Y. Hauser F. McCammon J. A. Rappel W. J.. (2018). Identification of SLAC1 anion channel residues required for CO_2_/bicarbonate sensing and regulation of stomatal movements. Proc. Natl. Acad. Sci. U.S.A. 115, 11129–11137. 10.1073/pnas.1807624115 30301791 PMC6217375

[B73] Zhang W. Jeon B. W. Assmann S. M. (2011). Heterotrimeric G-protein regulation of ROS signalling and calcium currents in *Arabidopsis* guard cells. J. Exp. Bot. 62, 2371–2379. 10.1093/jxb/erq424 21262908

[B74] Zhang W. Nilson S. E. Assmann S. M. (2008). Isolation and whole-cell patch clamping of *Arabidopsis* guard cell protoplasts. CSH Protoc. 2008:pdb.prot5014. 10.1101/pdb.prot5014 21356848

[B75] Zhang X. Zhang L. Dong F. Gao J. Galbraith D. W. Song C. P. (2001). Hydrogen peroxide is involved in abscisic acid-induced stomatal closure in Vicia faba. Plant Physiol. 126, 1438–1448. 10.1104/pp.126.4.1438 11500543 PMC117144

