# Supplementary material for: The Cyclophilin ROC3 Regulates ABA-Induced Stomatal Closure and the Drought Stress Response of Arabidopsis thaliana
Source: Front Plant Sci. 2021 May 25;12:668792. doi: 10.3389/fpls.2021.668792 (PMC8186832; doi:10.3389/fpls.2021.668792)
Supplement: Supplementary Table 1 — Primer sequences used in the experiments. [file Table_1.DOCX]

**Table S1** Primer sequences.

| **Name** | **Forward (5'-3')** |
| --- | --- |
| roc3-1-LP | CCGAAAACACACGAACGATAG |
| roc3-1-RP | ACTGAGATCCATTCGTGTTCG |
| roc3-2-LP | CCGAAAACACACGAACGATAG |
| roc3-2-RP | ACTGAGATCCATTCGTGTTCG |
| LBb1.3 | ATTTTGCCGATTTCGGAAC |
| ROC3-GUS-F | CCAAGCTTGGCTTCTCACATTTATTGCTTG |
| ROC3-GUS-R | CCCCCGGGGGTTCTTTTTTTCTCTGAGATTGATTG |
| ROC3-GFP-F | CGGGATCCATGGCAACAAACCCTAAAGT |
| ROC3-GFP-R | CGGTCGACAGAAATCTGACCACAATCAG |
| ACTIN2-qRT-F | GGTAACATTGTGCTCAGTGGTGG |
| ACTIN2-qRT-R | AACGACCTTAATCTTCATGCTGC |
| ROC3-qRT-F | TGCGAACACGAATGGATCTC |
| ROC3-qRT-R | CCTCAACAACTTGACCAAACAC |
| RBOHD-qRT-F | TCAACAACATGAAAGGTCC |
| RBOHD-qRT-R | CTAGAAGTTCTCTTTGTGG |
| RBOHF-qRT-F | CAGCAACCGCCATTAATG |
| RBOHF-qRT-R | CATCGAACAGTTCCAATGC |
| CAT1-qRT-F | TCAAATGCCTGTCGGATGAG |
| CAT1-qRT-R | GAAGAGATTCCACTGCGGATAG |
| CAT2-qRT-F | CTTTACACCAGAGAGGCAAGAA |
| CAT2-qRT-R | TCAGCCTGAGACCAGTAAGA |
| RD29A-qRT-F | GATGTTTAGGAAAGTAAAGGCTAG |
| RD29A-qRT-R | TAATCGGAAGACACGACAGG |
| RD29B-qRT-F | GGAGTGAAGGAGACGCAACAAG |
| RD29B-qRT-R | GGAATCCGAAAACCCCATAGTC |
| RAB18-qRT-F | CAGCAGCAGTATGACGAGTA |
| RAB18-qRT-R | CAGTTCCAAAGCCTTCAGTC |
| ABI5-qRT-F | CAATAAGAGAGGGATAGCGAACGAG |
| ABI5-qRT-R | CGTCCATTGCTGTCTCCTCCA |
| ABF2-qRT-F | TTGGGGAATGAGCCACCAGGAG |
| ABF2-qRT-R | GACCCAAAATCTTTCCCTACAC |
| ABF3-qRT-F | CTTTGTTGATGGTGTGAGTGAG |
| ABF3-qRT-R | GTGTTTCCACTATTACCATTGC |
| ERD10-qRT-F | TCTCTGAACCAGAGTCGTTT |
| ERD10-qRT-R | CTTCTTCTCACCGTCTTCAC |
| COR47-qRT-F | GAAAAGCTTCACCGATCCAA |
| COR47-qRT-R | TACCGGGATGGTAGTGGAAA |
| ROC3-C-F | CCCCCGGGGGATGGCAACAAACCCTAAAGT |
| ROC3-C-R | CGAGCTCGCTAAGAAATCTGACCACAATCAG |
