# Supplementary figures and images for: The Cyclophilin ROC3 Regulates ABA-Induced Stomatal Closure and the Drought Stress Response of Arabidopsis thaliana
Source: Front Plant Sci. 2021 May 25;12:668792. doi: 10.3389/fpls.2021.668792 (PMC8186832; doi:10.3389/fpls.2021.668792)

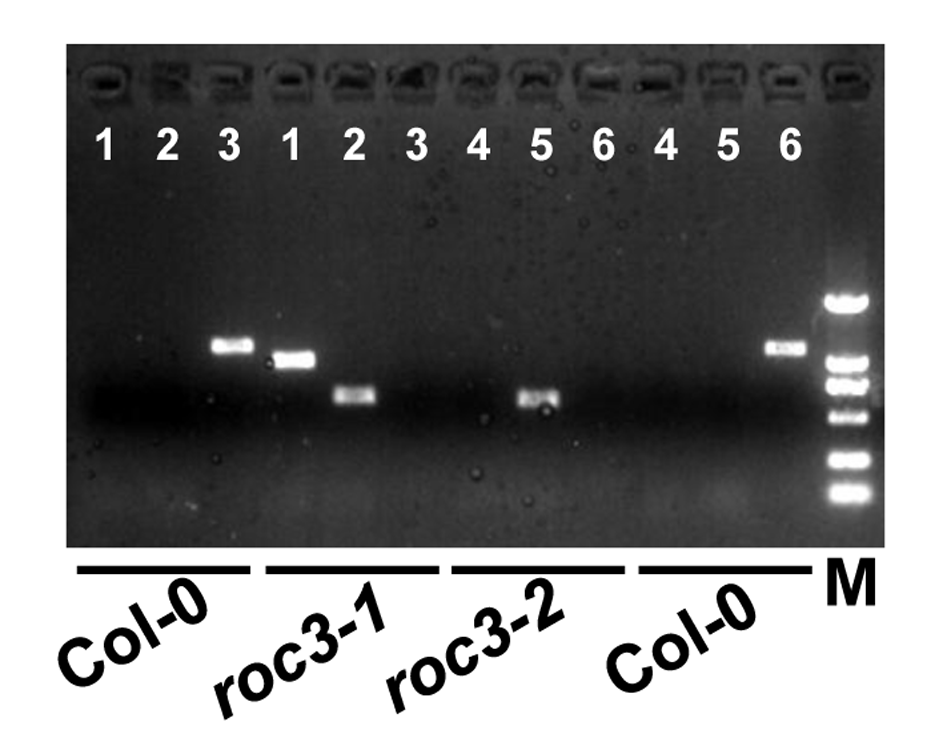

Supplement: Supplementary Figure 1 — PCR screening of homozygous T-DNA mutants of ROC3. 1: roc3-1-LP+LBb1.3; 2: roc3-1-RP+LBb1.3; 3: roc3-1-LP+ roc3-1-RP; 4: roc3-2-LP+LBb1.3; 5: roc3-2-RP+LBb1.3; 6: roc3-2-LP+ roc3-2-RP; M: Marker. [file Image_1.TIF]

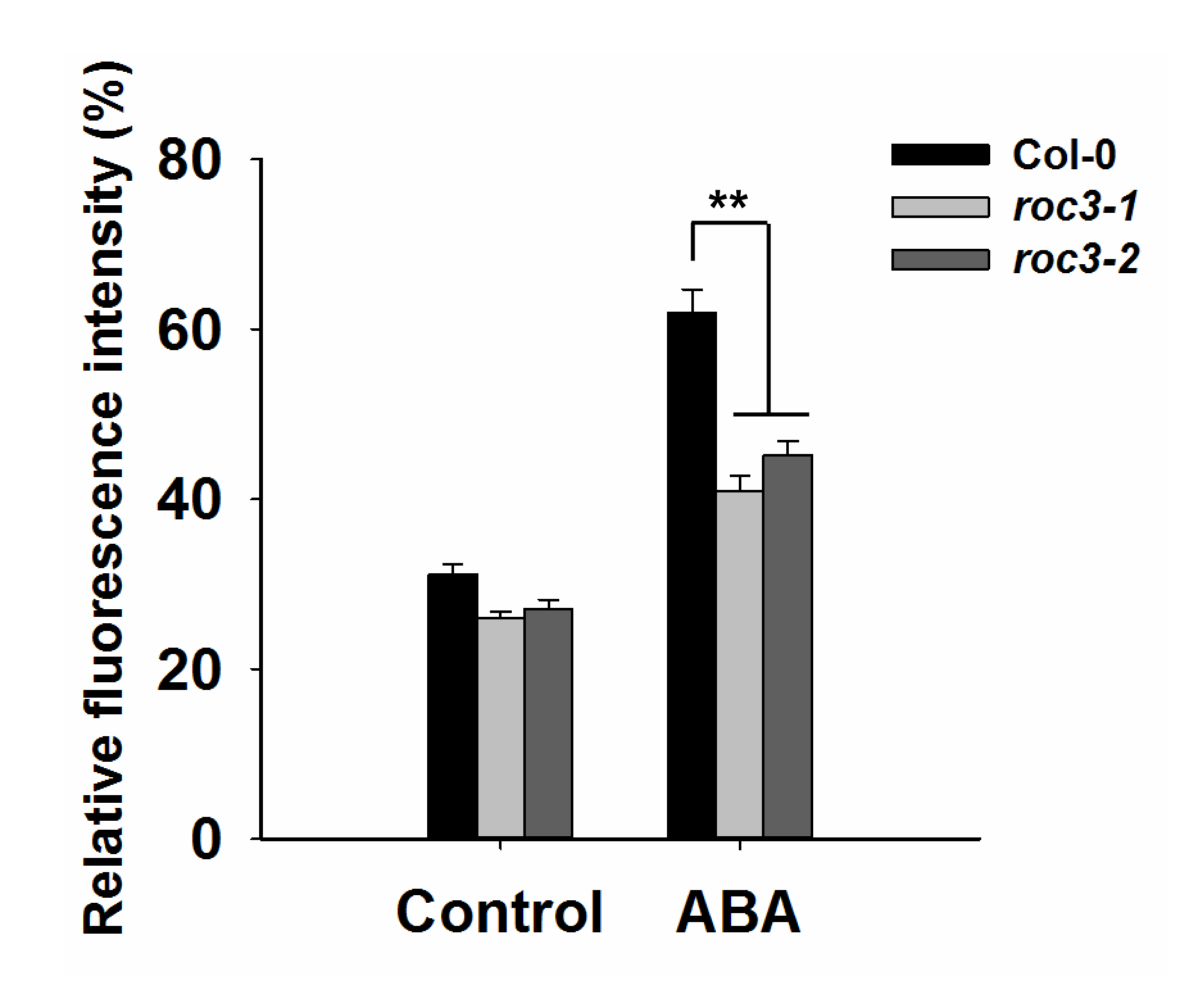

Supplement: Supplementary Figure 2 — Fluorescence micrographs revealing the ROS content of guard cells sampled from WT or roc3 mutant plants either exposed or not exposed to ABA; the fluorescent signal is generated from CM-H2DCFDA and captured by a conventional fluorescence microscope. There are 60 guard cells were sampled from each genotype. Error bars represent the SE (n = 60), **: means differed significantly from Col-0 (P <0.01). [file Image_2.TIF]

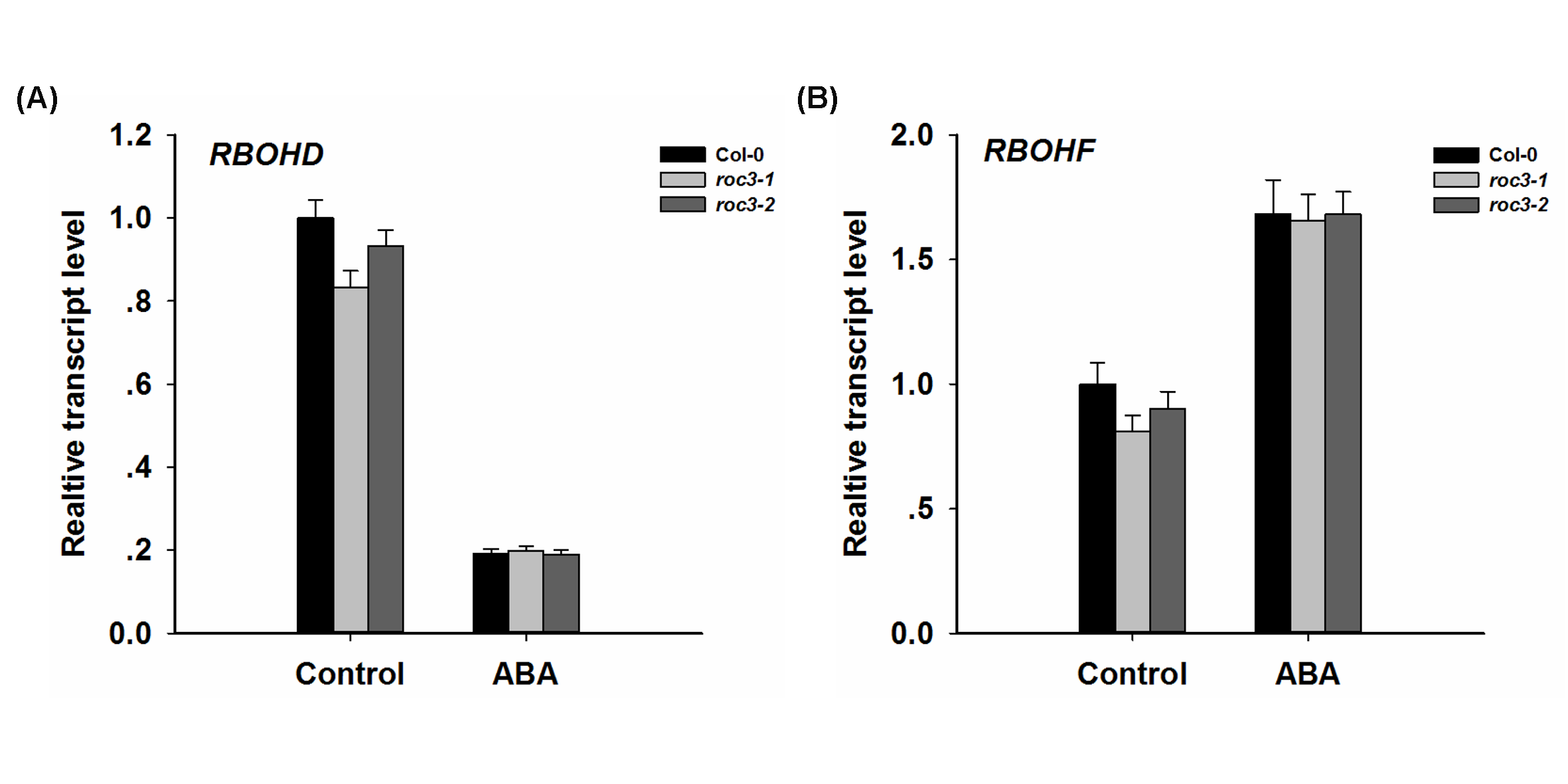

Supplement: Supplementary Figure 3 — Transcriptional profiling of two genes encoding NADPH/respiratory burst oxidase proteins. Relative transcript abundances of (A) RBOHD, (B) RBOHF, assessed using qRT-PCR, in WT or roc3 mutants either exposed or not exposed to ABA. Values shown in the form mean ± SE (n = 3). [file Image_3.TIF]
